# Global, regional, and national burden of cardiovascular diseases attributable to high body mass index from 1990 to 2021

**DOI:** 10.3389/fcvm.2025.1641689

**Published:** 2025-09-12

**Authors:** Liangtao Yao, Wenying Hou, Yan Zheng, Guohai Su

**Affiliations:** ^1^ Research Center of Translational Medicine, Central Hospital Affiliated to Shandong First Medical University, Jinan, Shandong, China; ^2^ School of Mathematics and Statistics, Shandong Normal University, Jinan, Shandong, China; ^3^ Department of Cardiovascular Medicine, Central Hospital Affiliated to Shandong First Medical University, Jinan, Shandong, China

**Keywords:** high body-mass index, cardiovascular disease, global burden of disease, epidemiology, risk factor

## Abstract

**Background:**

In recent decades, the escalating prevalence of obesity has contributed to a significant increase in the global burden of disease, with cardiovascular diseases (CVDs) emerging as the leading cause among all diseases attributable to high body-mass index (BMI). Utilizing global burden of disease (GBD) dataset from 1990 to 2021, we conducted a comprehensive analysis of the global, regional, and national trends in deaths and disability-adjusted life years (DALYs) attributable to CVDs caused by high BMI. Age-standardized mortality rate (ASMR) and age-standardized DALY rate (ASDR) were also investigated. Furthermore, we examined the associations of gender, age, and socio-demographic index (SDI) with the burden of CVDs attributable to high BMI. Finally, we assessed the evolution of health inequalities across countries and projected the global deaths and DALYs due to high BMI-related CVDs over the next two decades.

**Methods:**

The absolute numbers and the rates of age-standardized death, Disability-Adjusted Life Years (DALYs) per 100,000 people due to high BMI-related CVDs between 1990 and 2021 were extracted from GBD 2021. The estimated annual percentage changes (EAPCs) of high BMI-related CVDs disease burdens were calculated under the GBD's comparative risk assessment framework. Additionally, the disease burden prediction of the high BMI-related CVDs from 2022 to 2041 was performed using the bayesian age-period-cohort (BAPC) statistical model.

**Results:**

In 2021, high BMI-related CVDs accounted for 1.90 million deaths globally, representing an increase of 120.63% compared to 1990, with DALYs rising by 115.47% over the same period. Notably, while ASMR and ASDR among male showed no decline, female experienced 11.30% reduction in ASMRs and 6.12% reduction in ASDR. The burden was disproportionately borne by middle-aged and older populations across all age groups. Global health inequalities related to high BMI-related CVDs demonstrated a narrowing trend from 1990 to 2010, followed by a reversal into a negative correlation and continued to widen until 2021. Looking ahead, the burden of high BMI-related CVDs is projected to rise significantly due to population growth, the increasing prevalence of obesity, and aging populations.

**Conclusion:**

The results indicate that from 1990 to 2021, the burden of CVDs caused by high BMI has significantly increased. Particular attention should be directed toward middle and low-middle SDI regions. To mitigate this burden, it is imperative to implement public health strategies that emphasize education and awareness regarding the correlation between high BMI and CVDs. Policies promoting healthy dietary habits and regular physical activity are essential for reducing the future impact of high BMI-related cardiovascular morbidity and mortality. Such measures are not only urgently needed but also offer substantial long-term benefits for global health.

## Introduction

Over the past three decades, the global overweight and obesity rates have risen significantly, posing a major challenge to public health (1, 2). The body mass index (BMI), proposed by the World Health Organization (WHO), measures the ratio of weight to height and is used to determine whether adults are underweight, normal weight, overweight, or obese (3). According to a study published in *The Lancet* last year (4), between 1990 and 2022, the rate of high BMI among children and adolescents worldwide nearly quadrupled. Almost all countries showed an upward trend. During the same period, the rate of high BMI among adult women more than doubling, while the rate among adult men more than tripling. By 2022, a total of 159 million children and adolescents and 879 million adults worldwide were affected by high BMI.

High BMI is closely associated with a wide range of diseases, including cardiovascular diseases (CVDs) (5, 6), malignant tumors (7), diabetes (8), osteoarthritis (9), and depression (10). Existing research indicates that CVDs are the leading cause of disease burden related to high BMI. For instance, Nejadghaderi et al., using data from the 2019 Global Burden of Disease (GBD) database, pointed out that CVDs are the primary contributors to the disease burden caused by overweight in the Middle East and North Africa (11). Similarly, a 2017 paper published in The New England Journal of Medicine by Ashkan Afshin et al., which analyzed data from 195 countries between 1990 and 2015, found that over two-thirds of deaths related to high BMI were caused by CVDs (12). More recently, Yuhan Chen et al., based on data from the 2019 GBD database, analyzed the disease burden related to high BMI in 204 countries and regions. They found that CVDs are the main cause of high BMI-related mortality and disability-adjusted life years (DALYs) (13). DALYs is a metric for quantifying the burden of disease, defined as the sum of years of life lost (YLL) due to premature mortality and years lived with disability (YLD). For example, if a person dies in a traffic accident at age 30 (standard life expectancy 80 years) and had lived for two years with paraplegia (disability weight 0.5), then YLL = 80 − 30 = 50 years, YLD = 2 × 0.5 = 1 year, and DALYs = 51 years. Additionally, our query of the GBD 2021 database showed that CVD has consistently been the main contributor to the disease burden attributable to high BMI, as illustrated in Supplementary Figure S1.

CVDs encompass a range of conditions, including ischemic and hemorrhagic disorders, which originate from a variety of pathophysiological mechanisms, notably hyperlipidemia, elevated blood viscosity, and atherosclerosis, among others (14, 15). These conditions are particularly notable due to their substantial impact on morbidity and mortality. As one of the predominant non-communicable diseases globally, CVDs continue to pose a substantial challenge to public health efforts. A research report on cardiovascular diseases jointly compiled by the National Heart and Lung Blood Institute (NHLBI) of the National Institutes of Health (NIH) and the Institute for Health Metrics and Evaluation (IHME) from the University of Washington, reveal a marked increase in global CVD-related mortality from 12.4 million deaths in 1990 to nearly 20 million in 2022 (16). This trend reaffirms CVDs as a leading contributor to the global disease burden. Moreover, the impact of CVDs extends beyond mortality, as these diseases are also associated with a significant disability rate. Approximately 75% of those impacted by CVDs suffer from work capacity impairment and cognitive dysfunction to varying degrees, significantly impacting their quality of life and social integration (17). Currently, research attributing specific disease burdens to high BMI has been reported, such as malignant tumors (18), diabetes (19), osteoarthritis (20), etc. Furthermore, although some previous studies have analyzed the overall disease burden caused by high BMI (1, 3, 13, 21), including CVDs, there is still a lack of specialized research on the CVDs burden attributable to high BMI.

For individuals, compared to risk factors associated with CVDs that require professional personnel and specialized equipment for measurement, such as blood pressure (22), low-density lipoprotein (23), and blood glucose (24), BMI is more readily accessible to the general public due to the simplicity of its measurement method and the availability of tools (25). The assessment of blood pressure, low-density lipoprotein, and blood glucose requires venepuncture and laboratory processing, whereas BMI can be obtained non-invasively with only an electronic scale and a wall-mounted height chart, making it a more readily accessible measurement in both clinical and community settings. Research on the CVDs burden caused by high BMI can facilitate the public in assessing their potential CVDs risk through their own BMI indices and thus adopt appropriate preventive measures to mitigate the risk of cardiovascular diseases, such as improving dietary habits and increasing physical activity. These preventive measures, in turn, can suppress risk factors such as hypertension and hyperlipidemia, creating a virtuous cycle that reduces the burden of CVDs. For stakeholders in the realm of healthcare policy-making, the insights gleaned from this study about the correlation between high BMI and the burden of CVDs can serve as a basis for formulating effective prevention and management strategies. These strategies are essential for easing the significant economic pressures exerted by CVDs attributable to high BMI on both individuals and society as a whole.

In this study, we extracted the most recent 2021 GBD data about the burden of CVDs attributable to high BMI, encompassing both mortality and DALYs, spanning from 1990 to 2021. We presented the trends in global CVDs burden attributed to high BMI in the form of charts, calculated the expected annual percentage change (EAPC) for quantitative analysis, and synthesized the results of attributable disease burden to illuminate disparities among different regions and countries. Furthermore, we took into account the influence of sociodemographic factors, including age, gender, and the Socio-Demographic Index (SDI), on the CVDs burden associated with high BMI. On this basis, we forecast the trends of CVDs burden attributable to high BMI over the coming two decades with the Bayesian Age-Period-Cohort (BAPC) model—now the gold-standard projection tool in GBD studies for simultaneously accounting for age, period, and cohort effects while handling data uncertainty.

## Methods

### Data

The data regarding the CVDs burden attributable to high BMI (including the number of deaths, DALYs, and corresponding ASRs) were obtained from the GBD 2021 database using the Global Health Data Exchange (GHDx) query tool, stratified by age, gender, location, and the Socio-demographic Index (SDI).

The GBD database, established under the leadership of the Institute for Health Metrics and Evaluation (IHME) at the University of Washington, integrates data from official government sources, hospital records, surveys, and scientific literature. These data, which are subjected to cleaning, standardization, and modeling to ensure their quality and comparability, are utilized to assess and analyze the health impact of diseases, injuries, and risk factors on a global and regional scale, covering a wide range of health indicators from 1990 to 2021 (or the most recent year of database update).

In GBD 2021, a total of 88 risk factors are documented and categorized into three major classes: environmental, behavioral, and metabolic. These risk factors are further divided into four hierarchical levels based on their inclusion relationships and causal networks. High BMI is classified as a second-level risk factor under the category of metabolic risks. Similarly, the causes of health burden are categorized into four hierarchical levels. CVDs are classified as a second-level cause of health burden and encompasses seven subcategories of diseases, including stroke, ischemic heart disease, hypertensive heart disease, aortic aneurysm, and others. This study focuses on the CVDs burden attributable to high BMI.

### Definitions and analysis

BMI is a commonly used indicator for assessing the degree of obesity. The WHO has established classification criteria for BMI. BMI is calculated by dividing an individual's weight in kilograms by the square of their height in meters (13). For example, if a person weighs 70 kg and is 1.75 m tall, the BMI is calculated as follows: BMI = 70/(1.75)^2^ ≈ 22.86. In this study, high BMI is defined as a BMI greater than 25 kg/m^2^ for adults (aged 20+ years), in accordance with the GBD methodology.

The Sociodemographic Index (SDI) is a composite indicator that reflects the development status of a country or region. It is determined by three core factors: the total fertility rate for women under 25 years old, the average educational attainment of the population aged 15 and above, and per capita income (26). The SDI value typically ranges from 0 to 1, with higher values indicating better development status. The SDI is crucial for assessing the development levels of countries and regions globally. In academic research, the SDI is frequently categorized into five strata, ranging from low to high, in order to facilitate a more straightforward comparison and analysis of the development status among different countries or regions, as illustrated in Table 1 below.

**Table 1:** SDI stratification including country/region counts.

SDI stratification	SDI score range	Description	Number of countries/regions
Low SDI	0–0.47	Represents countries or regions with the lowest level of development	41
Lower-middle SDI	0.48–0.62	Represents countries or regions with a relatively low level of development, but slightly higher than those in the Low SDI category	41
Middle SDI	0.63–0.71	Represents countries or regions with a moderate level of development	40
High-middle SDI	0.72–0.81	Represents countries or regions with a relatively high level of development, but not yet reaching the highest level	41
High SDI	0.82–1	Represents countries or regions with the highest level of development	41

In this study, the number of deaths is examined as the total count of deaths within a year that are attributable to CVDs caused by high BMI. DALYs represent to the loss of healthy life years caused by premature death and disability due to CVDs attributable to high BMI. The corresponding age-standardized rates for these two metrics, namely the age-standardized mortality rate (ASMR) and the age-standardized disability-adjusted life years rate (ASDR), are calculated to eliminate the confounding effect of differences in age structure in the population based on the following formula.

$$\mathrm{ASR}=\frac{\sum_{i=1}^{A}a_{i}w_{i}}{\sum_{i=1}^{A}w_{i}}\times 100,000$$

In the formula above, *a_i_* represents the age-specific rate in the specific age group, *w_i_* represents the number or weight of individuals in the same specific age group from the chosen standard population, and *A* represents the total number of age groups. The ASMR and ASDR indicate the number of deaths and DALYs caused by diseases or injuries per 100,000 people adjusted for differences in age structure across populations. To illustrate the calculation of the ASR, consider a hypothetical country with 10 million inhabitants in 2024. Stratified by age, 15% are 0–14 years old and experience 30 cardiovascular deaths, 60% are 15–64 years old with 1,000 deaths, and 25% are 65 years or older with 2,000 deaths. When age-standardized to the World Standard Population, the ASMR for CVD falls to 20 per 100,000, well below the crude rate of 30.3.

Estimated annual percentage changes (EAPCs) and 95% confidence intervals (CIs) are computed to depict the trend of ASMR and ASDR of CVDs attributable to high BMI from 1990 to 2021 based on the following formula.

EAPC=100×(exp(β)−1)

Here, *β* is derived from a linear regression model that fits the year (*x*) with the logarithmically transformed age-standardized rate (ln(ASR)), expressed as ln(ASR) = *α* + *βx* + *ε*, where *β* represents the regression coefficient (27). If the lower bound of the 95% CI for the EAPC is greater than zero, it indicates that the ASR increased over the observation period. Conversely, if the upper bound of the 95% CI for EAPC is less than zero, it suggests a decreasing trend. When the 95% CI encompasses both positive and negative values, the ASR is considered to remained relatively stable. In this study, we used 95% CI because it optimally balance reliability and precision, satisfy conventional statistical significance thresholds, and align with international research practices.

The Slope Index of Inequality (SII) is a standardized indicator for measuring absolute gradient health inequality. We calculated the SII separately by regressing national ASMR and ASDR across all age groups on an SDI-associated relative position scale, which was defined by the midpoint of the cumulative range of population ranked by the SDI. We examined heteroscedasticity using a weighted regression model (21). Globally, the SII enables cross-country comparisons to pinpoint regions with pronounced health inequities. A negative SII indicates that the disease burden under study is more prevalent in countries with lower SDI, a value of zero indicates no difference in disease burden among countries, and a positive SII suggests a higher disease burden in countries with higher SDI.

To forecast the burden of CVDs attributable to high BMI from 2022 to 2046, we utilized the Bayesian Age-Period-Cohort (BAPC) model. This approach combines Bayesian statistical methods with the Age-Period-Cohort (APC) structure (28). The BAPC model employs a Bayesian approach that handles uncertainty, incorporates prior knowledge, and simultaneously accounts for the interactive effects of age, period, and cohort, thereby delivering more reliable projections and credible intervals. Compared with traditional APC model, it offers superior data handling and greater robustness of results, making it well suited for complex disease-burden analyses. By integrating prior information with observed data and applying second-order random walks to smooth the effects of age, period, and cohort, the BAPC model facilitates precise estimation of parameters and prediction of future disease burden trends (29). All analyses and visualizations were conducted using R software (version 4.4.1) and Origin (version 2024b), with statistical significance defined at *P* < 0.05.

## Results

### Global burden of CVDs attributable to high BMI

**Figure 1:**
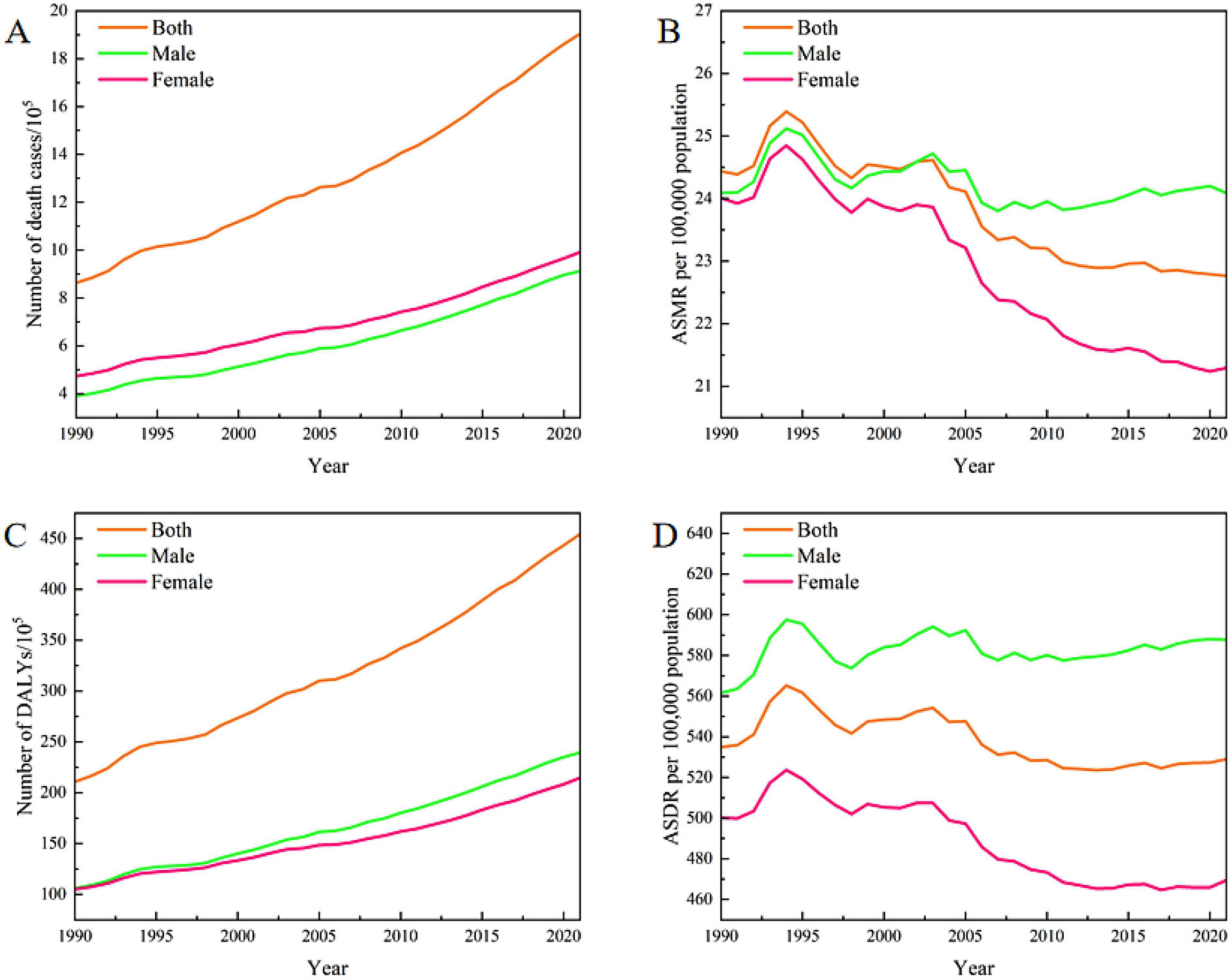
The numbers and rates of deaths and DALYs attributable to high BMI-related CVDs globally from 1990 to 2021.

**Figure 2:**
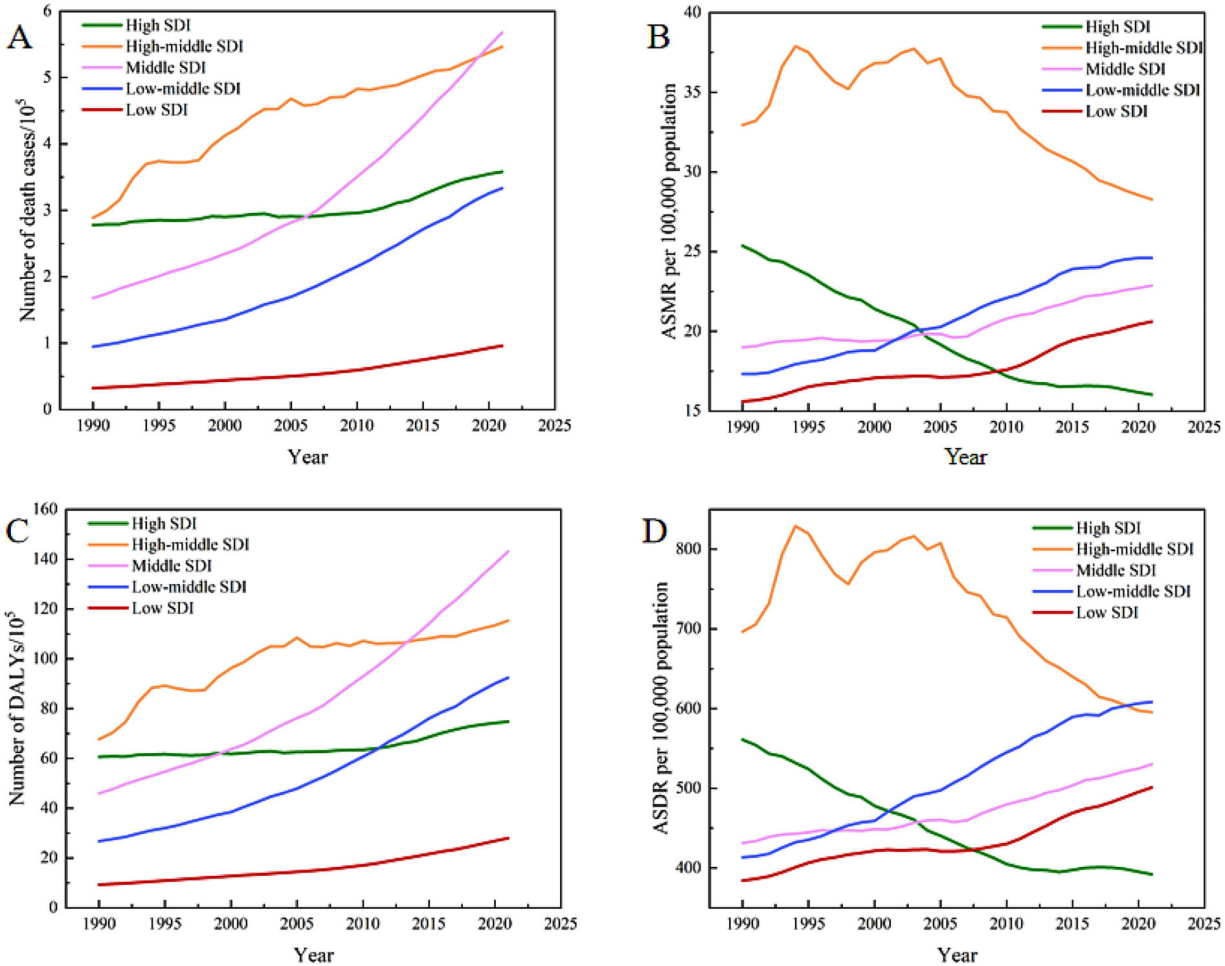
The numbers and rates of deaths and DALYs attributable to high BMI-related CVDs for the five SDI regions from 1990 to 2021.

**Figure 3:**
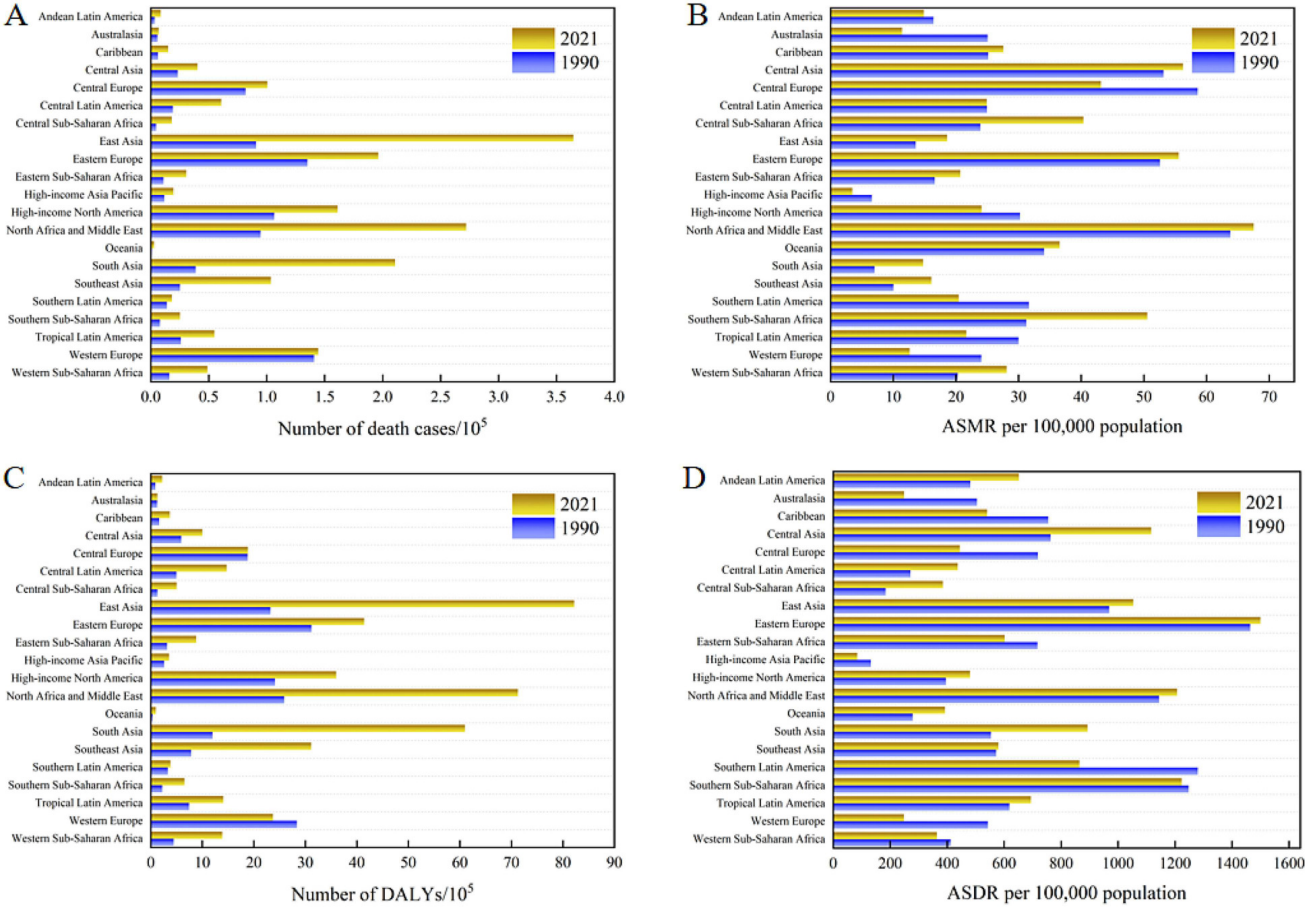
The numbers and rates of deaths and DALYs attributable to high BMI-related CVDs for the 21 GBD regions in 1990 and 2021.

In 2021, the number of deaths globally due to CVDs attributable to high BMI was 1.90 million (95% UI: 1.07, 2.86 million), representing a significant increase of 120.63% compared to 0.86 million (95% UI: 0.48, 1.32 million) in 1990 (Table 2 and Figure 1A). The corresponding ASMR decreased from 24.43 per 100,000 (95% UI: 13.63, 37.30) to 22.77 per 100,000 (95% UI: 12.87, 34.24), indicating a downward trend, confirmed by the negative EAPC of −0.35 (95% CI: −0.41, −0.30) (Table 1 and Figure 1B). Due to the large volume of data to be presented, for clearer visualization, we applied component labels by dividing the original data by 10^5^. A similar approach was also used in Figures 2, 3, 5, 7. The calculation method and interpretation of the EAPC are described in the Methods section. DALYs attributable to CVDs caused by high BMI also showed a similar trend, with the total DALYs increasing from 21.08 million (95% UI: 11.33, 32.46 million) in 1990 to 45.43 million (95% UI: 23.77, 69.60 million) in 2021, a rise of 115.47% (Table 3 and Figure 1C). The ASDR decreased from 535.01 per 100,000 (95% UI: 291.22, 821.15) to 528.99 per 100,000 (95% UI: 277.28, 808.64), with an EAPC of −0.19 (95% CI: −0.25, −0.13) (Table 2 and Figure 1D). The global burden of CVDs attributable to high BMI has been driven higher by population aging and increasing prevalence of obesity. The ASMR and ASDR for males did not decline, whereas for females, the ASMR and ASDR decreased by 11.30% and 6.12%, respectively, with corresponding EAPCs of −0.54 (95% CI: −0.61, −0.48) and −0.39 (95% CI: −0.46, −0.32).

**Table 2:** The deaths and ASMR attributable to high BMI-related CVDs in 1990/2021 and temporal trends of ASMR for the global, SDI regions, and GBD regions.

Regions	Number of death cases, 1990 (95%UI)	ASMR per 100,000 population, 1990 (95%UI)	Number of death cases, 2021 (95%UI)	ASMR per 100,000 population, 2021 (95%UI)	EAPC in ASMR per 100,000 population, 1990–2021 (95%CI)
Global	863,065.63 (477,898.20, 1,316,550.93)	24.43 (13.63, 37.30)	1,904,238.92 (1,072,732.31, 2,864,241.54)	22.76 (12.87, 34.24)	−0.35 (−0.41, −0.30)
Sex					
Male	389,736.01 (208,189.81, 598,500.61)	24.09 (13.11, 36.99)	912,341.17 (478,972.50, 1,393,234.19)	24.09 (13.20, 36.57)	−0.08 (−0.13, −0.04)
Female	473,329.61 (268,693.51, 724,371.99)	24.01 (13.45, 36.84)	991,897.75 (566,163.58, 1,486,812.53)	21.29 (12.11, 31.89)	−0.54 (−0.61, −0.48)
SDI region					
High SDI	277,601.30 (132,419.46, 453,026.12)	25.37 (12.02, 41.30)	358,201.45 (198,067.89, 548,404.81)	16.03 (9.08, 24.47)	−1.62 (−1.74, −1.50)
High-middle SDI	288,824.22 (140,671.30, 460,612.74)	32.95 (15.91, 52.22)	546,184.54 (277,704.01, 863,727.67)	28.29 (14.37, 44.72)	−0.77 (−0.99, −0.54)
Middle SDI	167,935.98 (113,575.04, 237,052.67)	19.00 (12.81, 26.79)	567,898.42 (322,611.15, 845,036.42)	22.87 (12.91, 34.13)	0.61 (0.52, 0.69)
Low-middle SDI	94,725.93 (60,102.31, 134,041.82)	17.33 (11.15, 24.52)	333,449.88 (193,977.54, 489,319.52)	24.60 (14.77, 35.86)	1.30 (1.25, 1.34)
Low SDI	32,179.72 (21,085.34, 43,123.92)	15.60 (10.36, 20.99)	95,845.76 (62,834.95, 134,646.38)	20.61 (13.64, 29.36)	0.83 (0.74, 0.92)
GBD region					
Andean Latin America	3,256.86 (1,793.64, 5,062.45)	16.43 (9.50, 25.05)	8,650.62 (4,687.25, 13,655.38)	14.90 (8.16, 23.54)	−0.26 (−0.46, −0.06)
Australasia	5,734.00 (2,356.86, 9,628.91)	25.08 (10.39, 42.26)	6,779.98 (3,166.02, 11,293.06)	11.42 (5.22, 18.93)	−2.71 (−2.83, −2.60)
Caribbean	6,225.16 (3,488.63, 9,089.04)	25.16 (14.20, 36.75)	15,010.65 (8,901.10, 22,646.47)	27.56 (16.26, 41.59)	0.43 (0.26, 0.60)
Central Asia	23,152.00 (10,106.77, 37,007.00)	53.14 (23.53, 84.75)	40,400.94 (19,535.02, 63,842.35)	56.25 (27.49, 89.36)	−0.17 (−0.56, 0.22)
Central Europe	81,722.84 (40,098.96, 131,704.62)	58.62 (28.74, 94.65)	100,600.68 (57,794.75, 154,547.52)	43.16 (24.92, 66.31)	−1.13 (−1.22, −1.04)
Central Latin America	18,922.82 (10,899.83, 28,642.55)	24.99 (14.88, 38.00)	60,771.81 (31,551.62, 94,872.99)	24.93 (13.01, 39.08)	−0.21 (−0.44, 0.02)
Central Sub-Saharan Africa	4,619.51 (2,923.24, 6,581.34)	23.91 (15.30, 34.01)	18,185.94 (10,862.77, 26,012.75)	40.35 (25.53, 59.34)	1.61 (1.57, 1.65)
East Asia	90,898.98 (64,317.00, 126,194.28)	13.62 (8.39, 19.68)	364,659.22 (194,223.00, 581,291.65)	18.60 (10.02, 29.02)	1.20 (1.00, 1.39)
Eastern Europe	135,165.13 (47,180.48, 229,422.29)	52.57 (18.42, 90.51)	196,338.91 (78,766.85, 327,894.41)	55.56 (22.28, 92.96)	−0.33 (−0.88, 0.22)
Eastern Sub-Saharan Africa	10,904.19 (7,205.15, 14,505.24)	16.63 (10.66, 23.38)	30,690.33 (21,259.72, 42,924.53)	20.72 (14.10, 29.08)	0.59 (0.51, 0.66)
High-income Asia Pacific	11,727.41 (6,900.45, 17,587.28)	6.60 (3.80, 9.97)	19,560.32 (10,436.72, 29,956.99)	3.47 (1.81, 5.32)	−1.98 (−2.31, −1.66)
High-income North America	106,552.40 (50,480.40, 174,891.60)	30.24 (14.40, 49.34)	161,236.87 (91,773.54, 242,298.44)	24.08 (13.78, 35.84)	−0.99 (−1.14, −0.84)
North Africa and Middle East	94,739.75 (55,886.86, 140,513.62)	63.79 (37.96, 94.05)	272,085.51 (153,283.79, 400,687.54)	67.54 (38.92, 100.73)	0.20 (0.11, 0.29)
Oceania	1,025.90 (538.08, 1,677.32)	34.07 (18.51, 55.35)	2,902.99 (1,411.23, 4,753.20)	36.57 (18.30, 59.43)	0.19 (0.15, 0.23)
South Asia	38,704.15 (23,186.87, 56,429.64)	7.04 (4.25, 10.11)	210,865.46 (115,245.36, 313,440.29)	14.79 (8.31, 22.09)	2.64 (2.54, 2.74)
Southeast Asia	25,130.64 (17,157.39, 34,614.99)	10.03 (6.83, 13.46)	103,564.09 (63,847.61, 152,176.10)	16.09 (10.17, 23.36)	1.58 (1.48, 1.68)
Southern Latin America	13,860.34 (7,191.38, 22,095.23)	31.66 (16.48, 50.44)	18,341.37 (10,427.68, 27,595.69)	20.44 (11.55, 30.74)	−1.09 (−1.20, −0.99)
Southern Sub-Saharan Africa	7,802.60 (5,361.35, 10,726.00)	31.25 (21.20, 43.42)	25,240.63 (16,766.04, 35,036.63)	50.54 (33.10, 71.27)	1.68 (1.21, 2.14)
Tropical Latin America	25,994.04 (14,304.92, 39,940.62)	30.01 (17.21, 45.94)	54,963.81 (31,080.31, 84,783.70)	21.68 (12.30, 33.27)	−1.01 (−1.06, −0.95)
Western Europe	140,805.87 (66,217.11, 227,542.24)	24.08 (11.30, 38.75)	144,491.93 (78,117.17, 225,161.21)	12.63 (6.75, 19.99)	−2.11 (−2.18, −2.05)
Western Sub-Saharan Africa	16,121.03 (10,935.89, 22,041.61)	20.30 (13.38, 27.77)	48,896.88 (30,108.12, 71,035.67)	28.10 (17.11, 40.94)	0.90 (0.76, 1.04)

Generated from data available from http://ghdx.healthdata.org/gbd-results-tool.

**Table 3:** The DALYs and ASDR attributable to high BMI-related CVDs in 1990/2021 and temporal trends of ASDR for the global, SDI regions, and GBD regions.

Regions	Number of DALYs, 1990 (95%UI)	ASDR per 100,000 population, 1990 (95%UI)	Number of death cases, 2021 (95%UI)	ASDR per 100,000 population, 2021 (95%UI)	EAPC in ASDR per 100,000 population, 1990–2021 (95% CI)
Global	21,082,656.72 (11,331,043.83, 32,461,612.85)	535.01 (291.22, 821.15)	45,426,878.84 (23,767,186.72, 69,603,287.54)	528.99 (277.28, 808.64)	−0.19 (−0.25, −0.13)
Sex					
Male	10,584,855.37 (5,472,051.82, 16,280,594.36)	561.58 (296.68, 865.26)	23,957,298.32 (12,186,757.93, 37,406,115.19)	587.75 (300.17, 913.39)	0.04 (−0.02, 0.09)
Female	10,497,801.35 (5,847,897.56, 16,145,528.56)	500.11 (279.25, 768.29)	21,469,580.52 (11,681,557.77, 32,032,441.46)	469.48 (254.30, 699.36)	−0.39 (−0.46, −0.32)
SDI region					
High SDI	6,061,548.20 (2,832,141.06, 9,729,481.20)	561.14 (262.29, 901.02)	7,481,117.63 (4,022,043.50, 11,418,018.72)	392.18 (208.92, 599.02)	−1.25 (−1.37, −1.13)
High-middle SDI	6,767,961.28 (3,125,856.04, 10,909,564.50)	696.53 (327.00, 1,119.01)	11,532,840.67 (5,403,645.04, 18,590,758.73)	595.53 (279.91, 958.54)	−0.89 (−1.16, −0.62)
Middle SDI	4,598,335.76 (2,946,816.21, 6,564,171.32)	431.50 (287.23, 613.19)	14,315,228.30 (7,452,578.33, 21,827,666.19)	530.25 (284.53, 798.72)	0.65 (0.59, 0.71)
Low-middle SDI	2,678,786.76 (1,658,780.03, 3,853,838.52)	413.21 (261.10, 588.14)	9,244,236.46 (5,209,810.94, 13,581,708.19)	608.36 (347.39, 888.55)	1.40 (1.35, 1.45)
Low SDI	932,976.11 (616,288.62, 1,270,322.09)	384.23 (254.52, 514.32)	2,795,043.30 (1,790,707.43, 3,953,351.69)	501.03 (325.37, 702.90)	0.75 (0.67, 0.84)
GBD region					
Andean Latin America	91,715.15 (46,412.32, 147,272.83)	411.82 (218.10, 652.07)	221,813.60 (110,198.84, 357,139.78)	364.65 (183.35, 586.48)	−0.39 (−0.60, −0.19)
Australasia	125,318.93 (50,455.37, 208,796.69)	542.70 (218.15, 902.86)	129,361.35 (57,338.58, 212,880.86)	248.67 (108.65, 408.01)	−2.67 (−2.82, −2.52)
Caribbean	163,530.25 (87,439.02, 241,894.03)	618.80 (333.01, 911.39)	372,304.93 (210,619.83, 562,112.95)	694.19 (391.59, 1,047.19)	0.50 (0.33, 0.67)
Central Asia	590,332.60 (247,462.42, 954,601.42)	1,247.88 (524.15, 2,015.54)	1,001,893.78 (461,567.66, 1,613,131.01)	1,223.31 (573.34, 1,950.20)	−0.55 (−0.97, −0.13)
Central Europe	1,881,701.64 (885,128.15, 3,055,812.31)	1,280.50 (602.65, 2,077.03)	1,885,399.06 (1,057,879.00, 2,924,068.27)	864.56 (483.14, 1,346.83)	−1.46 (−1.56, −1.37)
Central Latin America	498,383.26 (263,728.73, 772,158.86)	572.50 (316.14, 879.27)	1,474,962.20 (727,346.06, 2,327,248.06)	580.29 (288.97, 913.78)	−0.19 (−0.44, 0.05)
Central Sub-Saharan Africa	129,358.58 (81,295.30, 183,124.71)	554.54 (358.83, 777.80)	503,241.12 (298,880.09, 732,971.72)	892.62 (538.65, 1,285.60)	1.44 (1.39, 1.48)
East Asia	2,321,958.97 (1,667,594.70, 3,288,831.65)	278.89 (195.03, 391.76)	8,218,277.82 (4,055,209.08, 13,358,558.58)	392.39 (197.58, 631.99)	1.30 (1.12, 1.48)
Eastern Europe	3,117,490.12 (1,096,166.54, 5,255,899.95)	1,144.05 (400.42, 1,929.40)	4,140,140.08 (1,642,521.13, 6,909,224.59)	1,207.62 (475.73, 2,015.27)	−0.44 (−1.04, 0.16)
Eastern Sub-Saharan Africa	315,749.31 (209,630.04, 413,922.89)	395.53 (270.72, 528.04)	882,359.68 (596,594.00, 1,201,610.08)	480.45 (330.93, 668.85)	0.46 (0.37, 0.54)
High-income Asia Pacific	257,813.37 (147,337.63, 394,686.56)	132.81 (75.94, 202.11)	355,589.72 (180,457.75, 557,572.73)	84.69 (40.79, 134.90)	−1.45 (−1.66, −1.23)
High-income North America	2,410,149.22 (1,120,413.11, 3,872,029.39)	717.07 (334.54, 1,150.76)	3,598,605.04 (2,016,989.78, 5,332,227.25)	602.11 (343.10, 879.10)	−0.71 (−0.83, −0.60)
North Africa and Middle East	2,589,132.24 (1,448,194.42, 3,873,128.84)	1,464.05 (850.77, 2,179.82)	7,129,545.50 (3,799,060.08, 10,548,494.91)	1,499.72 (823.18, 2,207.62)	0.05 (−0.01, 0.10)
Oceania	34,888.31 (17,470.78, 57,339.17)	969.43 (503.26, 1,589.26)	98,570.43 (46,038.11, 163,498.50)	1,053.29 (504.47, 1,728.29)	0.24 (0.19, 0.29)
South Asia	1,197,401.00 (710,548.53, 1,755,954.32)	184.29 (110.85, 269.01)	6,096,089.72 (3,097,737.70, 9,141,485.06)	385.43 (200.83, 575.84)	2.63 (2.54, 2.72)
Southeast Asia	785,105.17 (518,959.69, 1,117,790.03)	271.70 (183.46, 378.90)	3,114,699.81 (1,795,106.87, 4,638,455.07)	437.64 (257.84, 646.45)	1.61 (1.50, 1.73)
Southern Latin America	329,756.60 (157,821.17, 529,621.70)	718.55 (346.92, 1,155.18)	382,274.03 (211,200.09, 580,594.60)	443.99 (242.31, 675.09)	−1.33 (−1.42, −1.25)
Southern Sub-Saharan Africa	221,780.42 (146,842.09, 304,340.67)	763.66 (511.49, 1,051.32)	654,860.74 (418,676.92, 914,457.46)	1,116.80 (720.36, 1,551.80)	1.37 (0.93, 1.82)
Tropical Latin America	742,559.74 (384,529.26, 1,169,441.01)	755.66 (404.20, 1,177.69)	1,406,607.01 (728,842.19, 2,181,502.59)	540.61 (281.65, 836.09)	−1.14 (−1.20, −1.09)
Western Europe	2,835,370.24 (1,293,889.87, 4,580,657.53)	504.53 (228.45, 814.60)	2,372,743.83 (1,252,565.18, 3,809,676.58)	249.09 (133.73, 398.31)	−2.35 (−2.42, −2.27)
Western Sub-Saharan Africa	443,161.61 (298,870.67, 621,753.42)	481.59 (329.16, 669.82)	1,387,539.40 (851,581.02, 2,036,083.63)	652.42 (400.59, 946.98)	0.84 (0.69, 0.98)

Generated from data available from http://ghdx.healthdata.org/gbd-results-tool.

### Burden of CVDs attributable to high BMI in SDI regions

In comparison to 1990, the GBD data of 2021 revealed a general rise in deaths and DALYs across all SDI regions. (Tables 2, 3, Figures 2A,C and Supplementary Figure S2). Specifically, the number of deaths increased by 0.40 million in middle SDI regions, 0.257 million in high-middle SDI regions, and 0.239 million in low-middle SDI regions over this period. The increases in deaths observed in high and low SDI regions were relatively modest, amounting to 0.081 million and 0.064 million, respectively. It is noteworthy that, despite having the smallest absolute increase in deaths among the five regions, the low SDI region demonstrated the highest percentage increase at 197.85%, outpacing the growth rates seen in both high and high-middle SDI regions. From 1990 to 2021, a decline in ASMR was observed in high and high-middle SDI regions, contrasting with an increase in ASMR across the other three SDI regions. Notably, the high SDI region experienced the largest decrease in ASMR at −36.83%, whereas the low-middle SDI region saw the highest increase at 41.96% (Figure 2B).

Over the past three decades, the trends in the number and rate of DALYs have been similar to those observed for deaths across all SDI regions (Table 2 and Figures 2C,D). In 2021, the total DALYs burden was highest in the middle SDI regions. The ASDR in low-middle SDI regions, after a period of rapid growth, reached 608.36 per 100,000 (95% UI: 347.39, 888.55) in 2021, which was the highest among the five SDI regions.

### Burden of CVDs attributable to high BMI across 21 GBD regions

In 2021, East Asia, North Africa and Middle East, and South Asia had the highest numbers of deaths attributable to high BMI-related CVDs, with South Asia experienced the fastest growth among the 21 GBD regions over this period by 4.45-fold and 4.09-fold, respectively (Table 2 and Figure 3A). Among the 21 GBD regions, Western Europe experienced the smallest increase in the death burden associated with high BMI-related CVDs. Furthermore, it was the only region to show a decline in DALYs, with a reduction of 16.32% (Tables 2, 3 and Figure 3C). During the period from 1990 to 2021, the ASMR in South Asia showed the fastest average annual percentage change, with an EAPC of 2.64 (95% CI: 2.54 to 2.74), which was the highest among all GBD regions. In 2021, the regions with the highest ASMR were North Africa and Middle East, Central Asia, and Eastern Europe. Australasia, Western Europe, and High-income Asia Pacific exhibited the most significant decreases in ASMR, with EAPCs of −2.71 (95% CI: −2.83, −2.60), −2.11 (95% CI: −2.18, −2.05), and −1.98 (95% CI: −2.31, −1.66), respectively (Table 1 and Figure 3B). The ASDR followed the same distribution and trend as ASMR across the 21 GBD regions. The highest ASDR values were observed in North Africa and Middle East, Central Asia, and Southern Sub-Saharan Africa, while High-income Asia Pacific was the lowest. South Asia also had the fastest growth rate in ASDR, with an EAPC of 2.63 (95% CI: 2.54, 2.72) (Table 2 and Figure 3D).

### Burden of CVDs attributable to high BMI across 204 countries and territories

**Figure 4:**
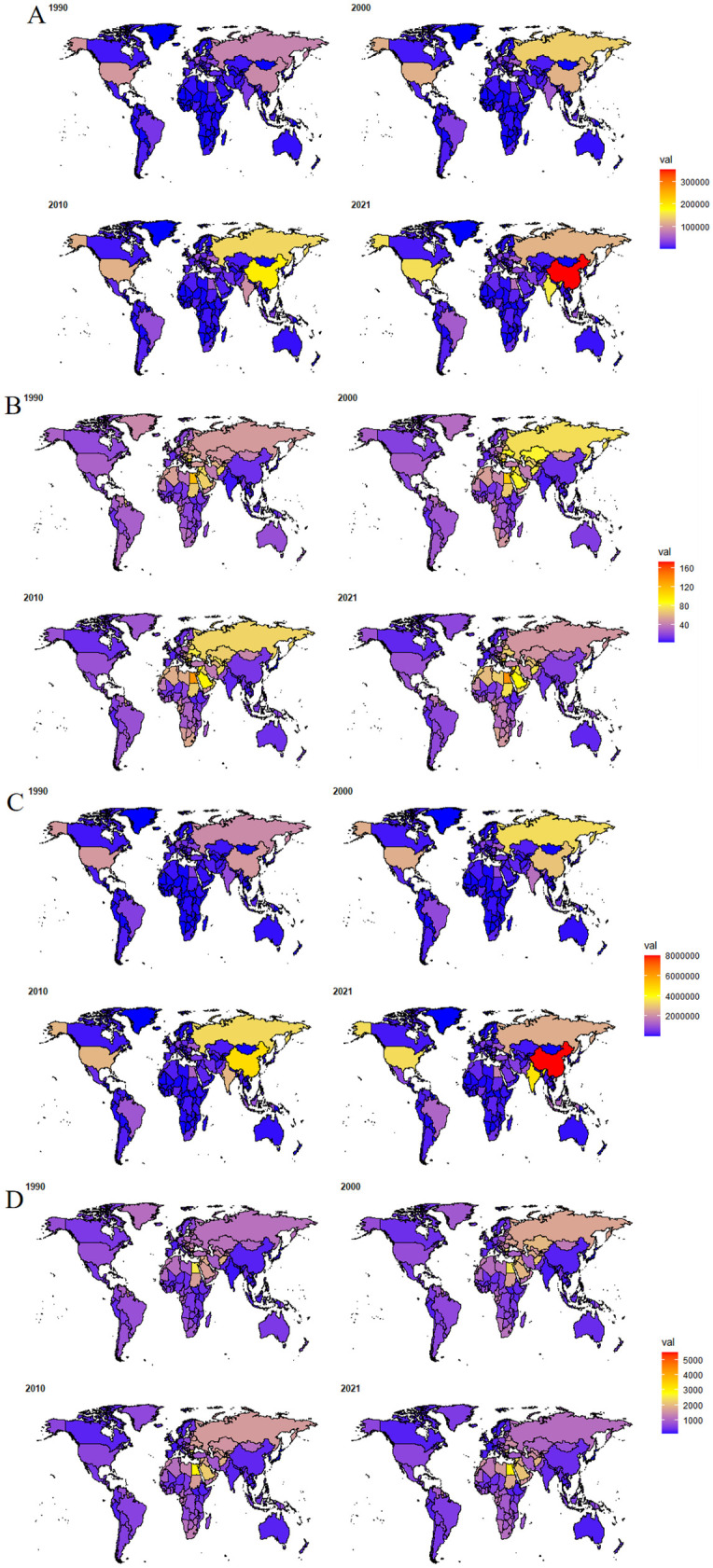
Distribution of **(A)** deaths, **(B)** ASMR, **(C)** DALYs and **(D)** ASDR attributable to high BMI-related CVDs for the 204 countries and territories in 1990, 2000, 2010 and 2021. The world map was drawn using the ggplot2 (30) package and the maps (31) package.

Figure 4 illustrates the distribution of deaths, DALYs, ASMR and ASDR attributable to high BMI-related CVDs across 204 countries and territories in 1990, 2000, 2010, and 2021. The disease burden is represented by colors ranging from purple (lightest) to yellow and then to red (heaviest). The burden of CVDs attributable to high BMI and its changes varied significantly between 1990 and 2021. In 2021, 191 and 176 countries experienced increases in the number of deaths and DALYs compared to 1990 (Supplementary Tables S1, S2 and Figures 4A,C). It is noted that national-level ASMR and ASDR remove the confounding effect of population growth over time, whereas absolute counts of deaths and DALYs retain the simultaneous influence of population growth and aging. The People's Republic of China, Republic of India, Arab Republic of Egypt, United States of America, and Republic of Indonesia had the largest increases in disease burden in terms of both deaths and DALYs. Specifically, the proportion of global deaths in China rose from 10.22% to 18.62% between 1990 and 2021, while India's increased from 3.37% to 8.51%, together accounting for 27.12% of the global deaths due to CVDs attributable to high BMI in 2021. The United Arab Emirates experienced the fastest growth rate in the death burden, with deaths attributable to high BMI-related CVDs in 2021 being more than five times those in 1990. Several countries, including the Republic of India, People's Republic of Bangladesh, Republic of Djibouti, Democratic Republic of Timor-Leste, Republic of Honduras, Republic of Indonesia, and Democratic People's Republic of Korea, witnessed more than a fourfold increase in deaths attributable to high BMI-related CVDs between 1990 and 2021. The trends in DALYs at the national level were quite similar to those of deaths.

The corresponding ASMR and ASDR varied greatly among countries (Supplementary Tables S1,S2 and Figures 4B,D), with the Republic of Nauru having the highest ASMR and ASDR, which were 46 times and 58 times higher than those of Japan, respectively, the country with the lowest ASR in 2021. Additionally, countries around the Mediterranean, such as Bulgaria, Egypt, and the Syrian Arab Republic, ranked high in ASMR and ASDR among the 204 countries and territories. In contrast, countries in East and Southeast Asia, such as Japan, South Korea, Thailand, and Singapore, as well as those in Western and Northern Europe, like France, Netherlands, Norway, and Denmark, had low levels of ASMR and ASDR. Compared with 1990, significant increases in ASMR and ASDR were observed in countries like India and Pakistan in South Asia, and the Philippines in Southeast Asia, while the largest declines were seen in countries like the United Kingdom, Denmark, Norway, and the Netherlands in Europe.

### Distribution and trends of the high BMI-related CVDs burden by age or gender

**Figure 5:**
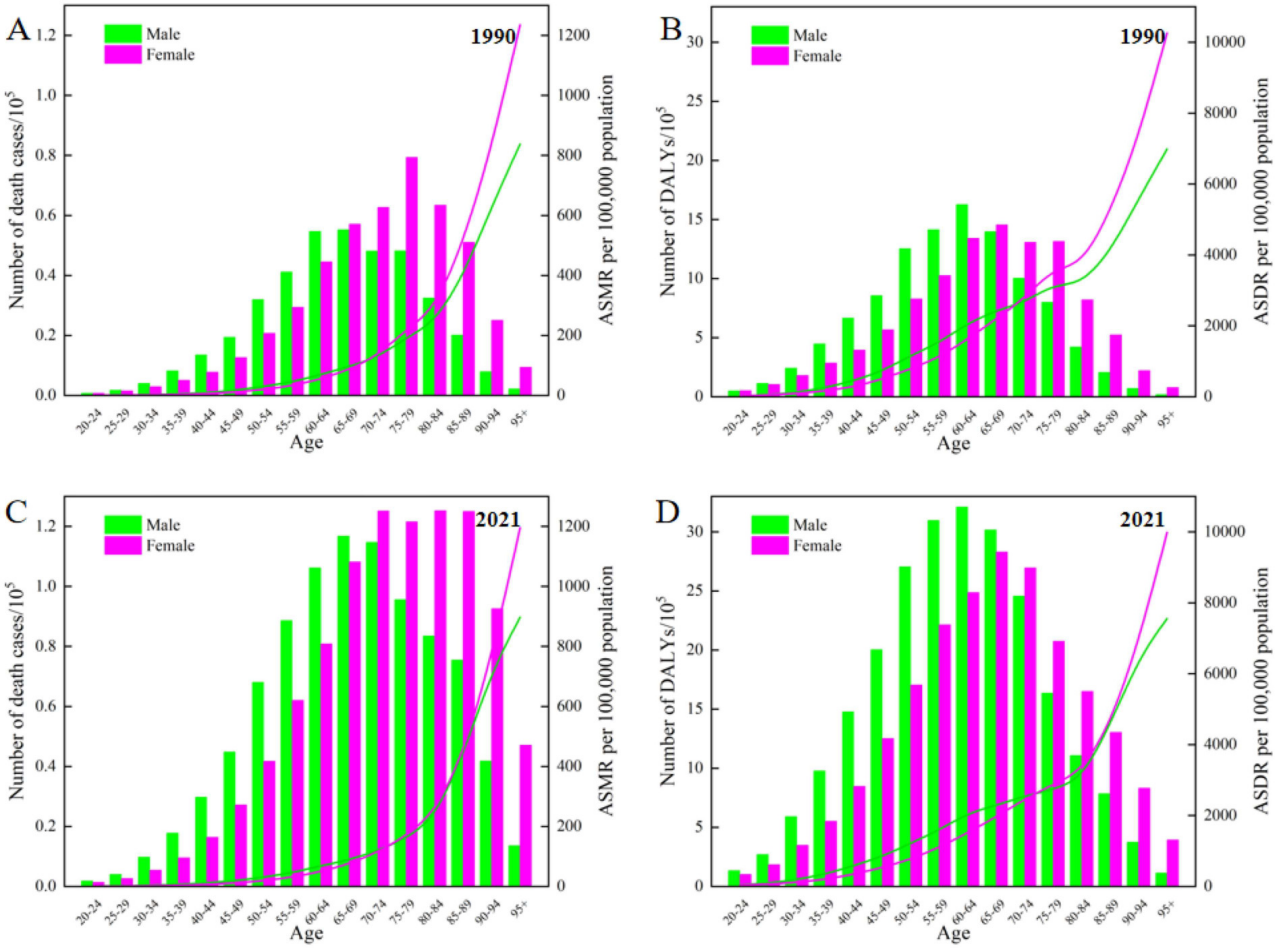
Age-specific numbers and rates of deaths and DALYs attributable to high BMI-related CVDs by gender in 1990 and 2021.

Figure 5 illustrates the numbers and rates of deaths and DALYs of CVDs caused by high BMI among different age groups by gender, the bar charts in the figure represent the number of deaths and DALYs, while the curves represent the corresponding ASR. Globally, in comparison to 1990, the mortality and DALYs counts associated with CVDs attributable to high BMI have significantly increased across all age groups for both genders in 2021 (Figure 5). Additionally, the corresponding ASMR and ASDR have exhibited significant growth similarly. During the youthful and middle-aged years, the burden of CVDs due to high BMI among males was higher than that of females. However, in the 65–69 age group, the disease burden for both males and females became comparable, and by the time they reached old age, the burden on females surpasses that of males. It is noteworthy that as age increased, the ASMR and ASDR for both males and females continued to rise, with a particularly rapid acceleration occurring after the age of 60.

In 1990, the ASMR for males and females was nearly equivalent, with males recording a rate of 24.09 (95% UI: 13.11, 36.99) and females at 24.01 (95% UI:13.45, 36.84). Over the following three decades, a significant decrease was observed in the ASMR for females, dropping to 21.29 (95% UI: 12.11, 31.89) in 2021. Conversely, the ASMR for males remained almost stable at 24.09 (95% UI: 13.20, 36.57), reflecting the rate documented in 1990. In terms of the ASDR, males have consistently demonstrated higher rates compared to females (Figure 1D). Throughout the entire study period, an upward trend has been evident for males. Between 1990 and 2021, the gap in ASDR between males and females has widened significantly, as illustrated in Figure 1D.

### Health inequality from 1990 to 2021 and projection of future high BMI-related CVDs burden

**Figure 6:**
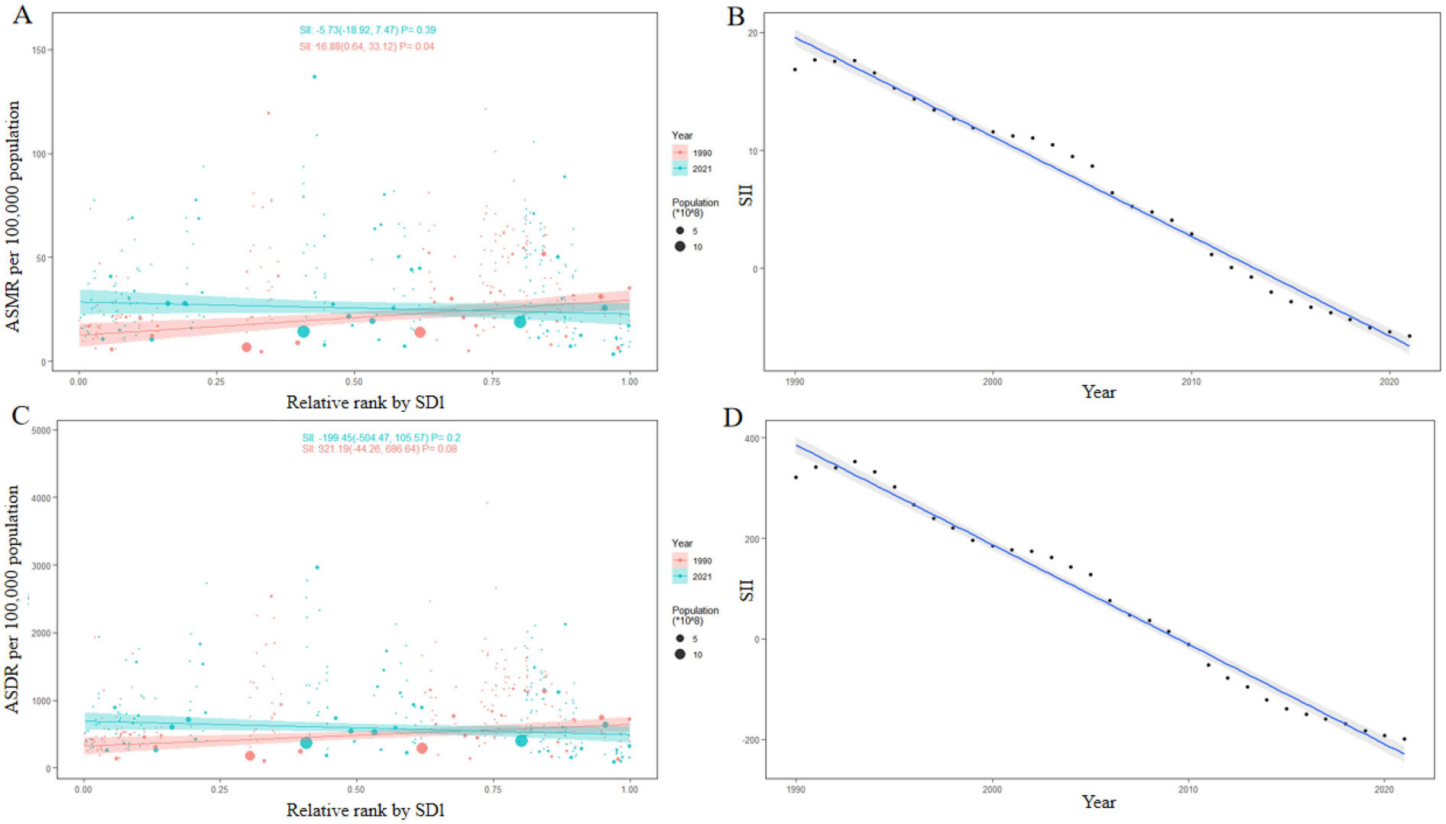
Health inequality regression curves of **(A)** ASMR and **(C)** ASDR in 1990 and 2021, and the trend of the SII of **(B)** ASMR and **(D)** ASDR from 1990 to 2021.

In 1990, the ASMR for CVDs attributable to high BMI across 204 countries and territories was 24.43 (95% UI: 13.63, 37.30), and the SII was 16.88 (95% UI: 0.64, 33.12) illustrated in Figure 6A. Details regarding the SII can be found in the Methods section. This suggested that country with the highest SDI exhibited an ASMR that was 16.88 higher compared to those with the lowest SDI. However, by 2021, the SII had shifted to −5.73 (95% UI: −18.92, 7.47), indicating a reversal in this situation. Over time, the absolute value of the SII had decreased between 1990 and 2021 as depicted in Figure 6B, signifying an improvement in health inequality. Nevertheless, upon examining the trend in the SII for the ASMR (Figure 6B), it had become evident that global health inequality had initially narrowed until 2010 but had widened since that time. This trend had indicated a deterioration in global health inequality over the past decade. Comparable conclusions can be drawn from the trends in health inequality reflected by the ASDR (Figures 6C,D).

**Figure 7:**
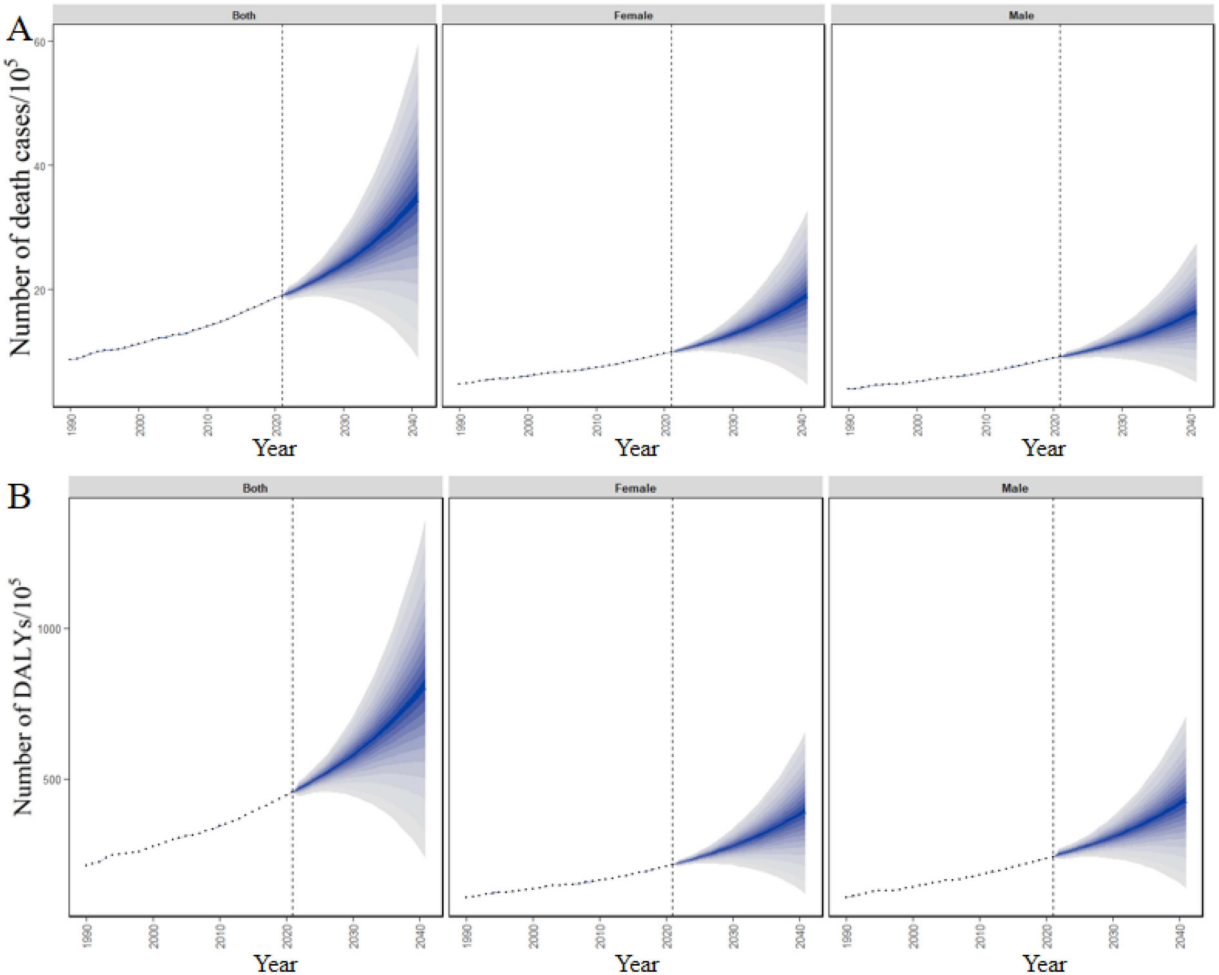
Future forecasts of number of **(A)** deaths and **(B)** DALYs attributable to high BMI-related CVDs globally from 2022 to 2041.

Figure 7 illustrated BAPC-derived global projections of deaths and DALYs from high-BMI-related CVDs over the next two decades, offering more reliable trends and credible intervals. According to the predictions, the number of deaths caused by high BMI-related CVDs globally will increase from 1.90 million in 2021 to 3.43 million in 2041. The predicted number of deaths is higher in females than in males, with a correspondingly greater growth rate (Figure 7A). Similarly, the DALYs attributable to high BMI-related CVDs globally are also projected to follow a similar trend, with an estimated increase of 76.31%. However, in contrast to the mortality trend, the DALYs are projected to be more severe in males than in females, as shown in Figure 7B.

## Discussion

### Overall global CVDs burden attributable to high BMI

In recent decades, the significant upward trend in high BMI globally has posed a major threat to global health (1). According to GBD 2021 on the burden of all kinds of diseases caused by high BMI at the global, regional, and national levels, CVDs ranked first for death, and second for DALYs in 2021 (Supplementary Figure S1). High BMI drives six CVD subtypes—ischemic heart disease, stroke, hypertensive heart disease, and three others—with ischemic heart disease accounting for the largest share of the burden (Supplementary Figure S3). Although the healthy burden caused by high BMI, whether in general or specifically for diseases such as cancer (7), diabetes (8), and joint disorders (9), has been investigated by researchers, to date, there remains a notable information gap concerning the detailed global, regional, and national levels of the burden of CVDs caused by high BMI. This burden is the main burden among all burdens attributable to high BMI, yet specific reports on it are scarce. This study presents the temporal trends and spatial distribution of the burden of CVDs caused by high BMI from 1990 to 2021 at the global, regional, and national levels using 2021 GBD data. It aids policymakers and individuals in understanding the high BMI-related CVDs burden that are relevant to their own interests, thereby providing a scientific basis for formulating effective prevention and intervention strategies and raising public awareness of the burden of CVDs caused by high BMI.

Between 1990 and 2021, the number of deaths and DALYs due to CVDs caused by high BMI increased significantly globally. In 2021, the number of deaths worldwide caused by high BMI-related CVDs reached a staggering 1.90 million, marking a 120.63% increase compared to 1990, accompanied by an 115.47% increase in DALYs. It is noteworthy that, despite the substantial surge in deaths and DALYs, the ASMR and ASDR associated with high BMI-related CVDs exhibited a slight downward trend. Specifically, the ASMR declined from 24.43 in 1990 to 22.77 in 2021, while the ASDR dropped from 535.01 in 1990 to 529.00 in 2021. Although this change may seem positive, considering the absolute increase in the number of deaths and DALYs, this decline may be more indicative of population aging and an expanding obese population rather than the control of high BMI-related CVDs burden. In terms of gender differences, the data from the 2021 GBD database reveals that males bear a heavier burden. Specifically, both in regard to ASMR and ASDR, males experience a higher burden than females. More worryingly, there has been no downward trend in males' ASMR and ASDR, which stands in stark contrast to the notable declines observed among females. Females have experienced 11.30% decrease in ASMR and 6.12% decline in ASDR, with corresponding negative EAPCs, indicating some progress in controlling the burden of CVDs among females. When examining the distribution of the burden of CVDs caused by a high BMI across different age groups, it is evident that males carry a heavier burden compared to females before age 65. Females experienced smaller rises in smoking, high-salt diets and physical inactivity than males, and consistently used community-based blood-pressure, lipid and BMI screening more in high-middle and high SDI countries. Behavioural gains were amplified by oestrogen-driven lipid modulation and the metabolic advantage of subcutaneous adiposity, as well as stronger social incentives to maintain slenderness, collectively yielding the pronounced decline in female CVD burden. Governments should pair aggressive tobacco and salt controls for males with sustained screening, weight-management incentives and equal cardiac care for females to reverse the growing male–female CVDs burden divide.

### Differences between regions

During the period from 1990 to 2021, deaths and DALYs in regions with different levels of SDI attributable to high BMI-related CVDs all showed a significant upward trend. The regions that experienced the most significant increase were those with middle SDI, and by 2021, both the number of deaths and DALYs caused by high BMI-related CVDs in these regions were the highest among the five different SDI regions, followed by the low-middle SDI region. Examining the ASMR and ASDR, regions with high and high-middle SDI saw a downward trend between 1990 and 2021, while the other three SDI regions exhibited an upward trend. The high SDI region experienced the largest decline, at −36.83%, while the low-middle SDI region saw the most significant increase, at 41.96%. Furthermore, the analysis of disease burden data across the 21 GBD regions shows that East Asia, North Africa and the Middle East, and South Asia experienced rapid increases in CVDs burden due to high BMI during the period from 1990 to 2021. These regions ranked in the top three among the 21 GBD regions in terms of total deaths and DALYs in 2021. This may be attributed to the prevalence of high BMI driven by economic development and urbanization, as well as evolving dietary habits and lifestyle changes in these regions (32, 33). In contrast to the aforementioned regions, Western Europe experienced the smallest increase in the burden of deaths caused by CVDs attributable to high BMI. Moreover, DALYs in this region decreased by 16.32%, making it the only region among the 21 GBD regions to witness a decrease, a fact also indicated by a negative EAPC. This is likely due to the well-developed public health systems, robust health education, and effective management of CVDs in Western Europe (34). In the 21 GBD regions, North Africa and the Middle East, Central Asia, Eastern Europe, and Southern Sub-Saharan Africa rank highest in ASMR and ASDR. From 1990 to 2021, CVDs burden attributable to high BMI rose more slowly in high-SDI regions than in low-SDI regions, as rapidly mounting CVDs burden driven by high-BMI in the latter has outpaced the capacity of its health systems. This indirectly highlights disparities in medical resources, health policies, and public health awareness in these regions. Across SDI levels, high and high-middle SDI regions should consolidate gains by extending fiscal incentives for reformulated foods and embedding weight management in universal health coverage. Middle SDI regions, experiencing the steepest increases, must urgently implement BMI-control policies and expand medical investment. Lower-middle and low SDI regions, facing rising ASMR amid limited resources, should offer brief obesity counseling and negotiate discounted drug prices to curb the growing disease burden.

### Disparities among countries

At the national level, significant disparities exist among countries and territories, shaped by economic and social development, lifestyle, healthcare quality, and population aging. Compared to 1990, 93.63% and 86.27% of countries and territories saw an increase in deaths and DALYs caused by high BMI-related CVDs, respectively. China and India, as populous nations, have been significant contributors to this rise. Over the past three decades, the obesity rates in India and China have shown a significant upward trend. This is mainly due to rapid economic development and improved living standards, which have provided people with more access to high-calorie and high-fat foods, while lifestyles have become more sedentary, lacking sufficient physical exercise (35, 36). During this period, the proportion of global deaths from CVDs attributable to high BMI in China increased from 10.22% in 1990 to 18.62% in 2021. In India, it rose from 3.37% to 8.51%. By 2021, the combined proportion of these two countries reached 27.12%. Additionally, significant differences among countries were driven by a range of factors. For example, the United States and Japan, both developed countries with advanced healthcare services, yet they showed different trends in the burden of CVDs attributable to high BMI. In the United States, this burden has continued to increase, while Japan has managed to curb it effectively. Overall, developed countries such as the Netherlands, France, Norway, Denmark in Western and Northern Europe, Japan and South Korea in East Asia, and Australia have mitigated the burden of CVDs caused by high BMI. This largely reflects the adoption of advanced technologies such as cardiac CT imaging, coronary artery calcium scoring (CAC) and CT coronary angiography (CCTA) for prevention and early detection of CVDs (37, 38), alongside robust health education and effective CVD management, in Western Europe and the High-income Asia Pacific region. China, along with India, Pakistan, Indonesia, the Philippines, Egypt, Brazil, and Russia, has been witnessing a rapid increase in the burden of CVDs attributable to high BMI. In contrast, some low-income countries in Africa and Southeast Asia, such as Sudan, Algeria, and Myanmar, have relatively lower obesity rates. However, due to limited medical resources and insufficient public health awareness, the burden of CVDs attributable to high BMI in these countries has significantly increased compared to 1990.

### Evolving health inequality in high BMI-related CVDs

Regarding the evolution of health inequality associated with high BMI-related CVDs, in 1990, countries with higher SDI exhibited significantly higher ASMRs and ASDRs, indicating pronounced health inequalities. A comparison of the SII between 1990 and 2021 reveals a decrease in its absolute value, suggesting that the health inequality gap may be narrowing. After closely examining the SII trends of ASMR and ASDR, we uncovered a more complex picture. The health inequality decreased steadily until around 2010, but then there was a reversal, with low SDI countries experiencing a higher burden of CVDs caused by high BMI than high SDI countries, and the gap between them continued to widen until 2021. This variation highlights the profound impact of socioeconomic factors on outcomes related to the burden of CVDs attributable to high BMI. The prediction of disease burden provides us with insights into the future trends of the burden of CVDs caused by high BMI. Over the next two decades, there will be a significant increase in deaths and DALYs attributable to CVDs caused by high BMI. Notably, the projected number of deaths among females is expected to not only exceed that of males but also increase at a faster rate. In terms of DALYs, gender differences are also notably evident. Although males will carry a greater burden of DALYs in the future, the rate of growth among females will be more dramatic.

### Suggestions for strategies

Governments and pertinent organizations should adopt location-specific strategies to improve the situation based on the differences in the CVDs burden caused by high BMI in various countries and regions. The most academically and socially influential cardiovascular prevention initiative ever launched—the North Karelia Project—began in Finland in 1972. It was implemented at the community level, and focused on reducing salt and saturated-fat intake, curbing tobacco use, and promoting physical activity. Over three decades, adult men's median BMI fell by roughly 2 kg/m^2^, serum cholesterol dropped by 20%, and coronary heart-disease mortality plummeted by 82% in men and 68% in women, driving a sustained nationwide decline in cardiovascular mortality across Finland. Drawing on this successful experience, they can implement population-weight-control measures and run awareness campaigns that highlight the link between high BMI and the burden of CVDs. This will encourage individuals to maintain a balanced diet, engage in regular exercise, and control their weight, thereby enhancing public awareness of CVDs prevention. At the same time, promoting a healthy lifestyle and continuously reducing the incidence of obesity can collectively help to lower the risk of CVDs.

### Strengths and limitations

The study has several strengths, including use the latest GBD data to specifically examine the changes and forecasts of the CVDs burden related to high BMI, which is the leading burden caused by high BMI, at the global, regional, and national levels from 1990 to 2021. However, it also has some limitations. First, the quality and availability of data vary significantly across different countries and regions. Many low and middle-income countries may have inadequate disease surveillance systems and lack advanced technical means and professional personnel for data processing and analysis, resulting in missing and inaccurate data. Second, while BMI is a simple, convenient, and inexpensive measure of obesity, it cannot reflect individual differences in body fat distribution and content (39). Third, the GBD data do not capture the CVDs burden among children and adolescents under 20 years old who are overweight or obese. Additionally, predictions regarding the future CVDs burden caused by high BMI are derived from existing trends and patterns. These projections do not account for possible shifts in lifestyles, improvements in therapeutic technologies, or alterations in healthcare policies that may occur in the future (40, 41). As such, these forecasts must be approached with caution, while remaining receptive to new data, research findings, and evolving viewpoints.

## Conclusions

In this study, we investigated the global, regional, and national burdens of CVDs attributable to high BMI. We analyzed the impacts of gender, age, and the SDI on this burden. Furthermore, we examined the evolution of health inequalities related to the burden of CVDs caused by high BMI worldwide and predicted the global burden of CVDs due to high BMI over the next two decades. The findings indicate that the burden of CVDs attributable to high BMI significantly increased between 1990 and 2021, with the overall burden was higher in males than in females until around the age of 65, after which it rapidly increased for both genders, but particularly so for females. Notably, there were significant decrease in ASMR and ASDR in high and high-middle SDI regions, while the burden of CVDs caused by high BMI increased in other SDI regions, with the highest burden observed in middle SDI regions. At the national level, significant variations were observed among different countries and territories due to factors such as varying levels of economic development, lifestyles, healthcare systems, and racial differences. The results emphasize the need for practical and effective solutions to raise public awareness about the prevention of CVDs caused by high BMI, against the backdrop of future population growth, obesity pandemics, and intensifying aging. Furthermore, promoting healthy lifestyles and increasing investments in medical resources are crucial for jointly reducing the incidence of high BMI and the burden of CVDs.

## Data Availability

The original contributions presented in the study are included in the article/Supplementary Material, further inquiries can be directed to the corresponding authors.

## References

[1] Wareham NJ Dai H Alsalhe TA Chalghaf N Riccò M Bragazzi NL The global burden of disease attributable to high body mass index in 195 countries and territories, 1990–2017: an analysis of the Global Burden of Disease Study. PLoS Med. (2020) 17(7):e1003198. 10.1371/journal.pmed.1003198 32722671 PMC7386577

[2] Al Ta'ani O Al-Ajlouni YA Aleyadeh W Al-Bitar F Alsakarneh S Saadeh A The impact of overweight and obesity on health outcomes in the United States from 1990 to 2021. Diabetes Obes Metab. (2024) 26(11):5455–65. 39261301 10.1111/dom.15924

[3] Wang P Huang S Wang R Shi X Xu H Peng J Global burden and cross-country inequalities in diseases associated with high body mass index from 1990 to 2019: result from the Global Burden of Disease Study 2019. J Glob Health. (2024) 14:04200. 39513280 10.7189/jogh.14.04200 PMC11544517

[4] Phelps NH Singleton RK Zhou B Heap RA Mishra A Bennett JE Worldwide trends in underweight and obesity from 1990 to 2022: a pooled analysis of 3663 population-representative studies with 222 million children, adolescents, and adults. Lancet. (2024) 403(10431):1027–50. 10.1016/S0140-6736(23)02750-2 38432237 PMC7615769

[5] Thaikruea L Thammasarot J. Prevalence of normal weight central obesity among Thai healthcare providers and their association with CVD risk: a cross-sectional study. Sci Rep. (2016) 6(1):37100. 10.1038/srep37100 27848990 PMC5111059

[6] Damigou E Chrysohoou C Barkas F Vlachopoulou E Vafia C Dalmyras D Mean body mass index during 20 years of follow-up and cardiovascular disease incidence: results from the ATTICA cohort study (2002–2022). Eur Heart J. (2024) 45(Suppl 1):ehae666-2880. 10.1093/eurheartj/ehae666.2880

[7] O’Sullivan J Lysaght J Donohoe CL Reynolds JV. Obesity and gastrointestinal cancer: the interrelationship of adipose and tumour microenvironments. Nature Reviews Gastroenterology & Hepatology. (2018) 15(11):699–714. 10.1038/s41575-018-0069-7 30323319

[8] Sassano M Castagna C Villani L Quaranta G Pastorino R Ricciardi W National taxation on sugar-sweetened beverages and its association with overweight, obesity, and diabetes. Am J Clin Nutr. (2024) 119(4):990–1006. 10.1016/j.ajcnut.2023.12.013 38569789

[9] Shumnalieva R Kotov G Monov S. Obesity-related knee osteoarthritis—current concepts. Life. (2023) 13(8):1650. 37629507 10.3390/life13081650 PMC10456094

[10] He K Pang T Huang H. The relationship between depressive symptoms and BMI: 2005–2018 NHANES data. J Affect Disord. (2022) 313:151–7. 10.1016/j.jad.2022.06.046 35753497

[11] Nejadghaderi SA Grieger JA Karamzad N Kolahi A-A Sullman MJM Safiri S Burden of diseases attributable to excess body weight in the Middle East and north Africa region, 1990–2019. Sci Rep. (2023) 13(1):20338. 37990049 10.1038/s41598-023-46702-y PMC10663478

[12] GBD 2015 Obesity Collaborators. Health effects of overweight and obesity in 195 countries over 25 years. N Engl J Med. (2017) 377(1):13–27. 10.1056/NEJMoa1614362 28604169 PMC5477817

[13] Chen Y Ma L Han Z Xiong P. The global burden of disease attributable to high body mass index in 204 countries and territories: findings from 1990 to 2019 and predictions to 2035. Diabetes Obes Metab. (2024) 26(9):3998–4010. 10.1111/dom.15748 38957939

[14] Yang C Wu Y Qian J Li J-J. A systematic, updated review of Xuezhikang, a domestically developed lipid-lowering drug, in the application of cardiovascular diseases. Acta Pharm Sin B. (2024) 14(10):4228–42. 10.1016/j.apsb.2024.05.011 39525586 PMC11544391

[15] Sharaf El Din UAA Salem MM Abdulazim DO. Uric acid in the pathogenesis of metabolic, renal, and cardiovascular diseases: a review. J Adv Res. (2017) 8(5):537–48. 10.1016/j.jare.2016.11.004 28748119 PMC5512153

[16] Mensah GA Fuster V Roth GA. A heart-healthy and stroke-free world: using data to inform global action. J Am Coll Cardiol. (2023) 82(25):2343–9. 10.1016/j.jacc.2023.11.003 38092508

[17] Stone KL Zhong J Lyu C Chodosh J Blachman NL Dodson JA. Does incident cardiovascular disease lead to greater odds of functional and cognitive impairment? Insights from the health and retirement study. J Gerontol Ser A. (2023) 78(7):1179–88. 10.1093/gerona/glad096 PMC10329231 36996314

[18] Ilic I Ilic M. Global burden of pancreatic cancer attributable to high body-mass index in 204 countries and territories, 1990–2019. Cancers (Basel). (2024) 16(4):719. 10.3390/cancers16040719 38398110 PMC10886782

[19] Zhang X Wang X Wang M Hu B Tang W Wu Y The global burden of type 2 diabetes attributable to high body mass index in 204 countries and territories, 1990–2019: an analysis of the global burden of disease study. Front Public Health. (2022) 10:966093. 10.3389/fpubh.2022.966093 36159296 PMC9500174

[20] Song M Chen H Li J Han W Wu W Wu G A comparison of the burden of knee osteoarthritis attributable to high body mass index in China and globally from 1990 to 2019. Front Med (Lausanne). (2023) 10:1200294. 10.3389/fmed.2023.1200294 37680622 PMC10481341

[21] Liu C Zhang Z Wang B Meng T Li C Zhang X. Global health impacts of high BMI: a 30-year analysis of trends and disparities across regions and demographics. Diabetes Res Clin Pract. (2024) 217:111883. 10.1016/j.diabres.2024.111883 39368489

[22] Allen NB Ning H Lloyd-Jones D. Abstract P070: long-term cumulative blood pressure improves CVD risk prediction algorithms. Circulation. (2016) 133(Suppl 1):AP070.

[23] Virani SS Aspry K Dixon DL Ferdinand KC Heidenreich PA Jackson EJ The importance of low-density lipoprotein cholesterol measurement and control as performance measures: a joint clinical perspective from the national lipid association and the American society for preventive cardiology. J Clin Lipidol. (2023) 17(2):208–18. 10.1016/j.jacl.2023.02.003 36965958

[24] Hong KN Fuster V Rosenson RS Rosendorff C Bhatt DL. How low to go with glucose, cholesterol, and blood pressure in primary prevention of CVD. J Am Coll Cardiol. (2017) 70(17):2171–85. 10.1016/j.jacc.2017.09.001 29050566

[25] Kranjac AW Kranjac D. Explaining adult obesity, severe obesity, and BMI: five decades of change. Heliyon. (2023) 9(5):e16210. 10.1016/j.heliyon.2023.e16210 37251838 PMC10213181

[26] Wu S Xu W Guan C Lv M Jiang S Jinhua Z. Global burden of cardiovascular disease attributable to metabolic risk factors, 1990–2019: an analysis of observational data from a 2019 global burden of disease study. BMJ Open. (2023) 13(5):e069397. 10.1136/bmjopen-2022-069397 37173115 PMC10186407

[27] Zou B Wu P Luo J Li L Zhou M. Analysis of the global burden of cardiovascular diseases linked to exposure to ambient particulate matter pollution from 1990 to 2019. Front Public Health. (2024) 12:1391836. 10.3389/fpubh.2024.1391836 39416944 PMC11479877

[28] Masaebi F Salehi M Kazemi M Vahabi N Azizmohammad Looha M Zayeri F. Trend analysis of disability adjusted life years due to cardiovascular diseases: results from the global burden of disease study 2019. BMC Public Health. (2021) 21(1):1268. 10.1186/s12889-021-11348-w 34187450 PMC8244206

[29] Zhang T Guo Y Qiu B Dai X Wang Y Cao X. Global, regional, and national trends in colorectal cancer burden from 1990 to 2021 and projections to 2040. Front Oncol. (2025) 14:1466159. 10.3389/fonc.2024.1466159 39886660 PMC11779618

[30] Becker RA Wilks AR. “Constructing a Geographical Database”, AT&T Bell Laboratories Statistics Research Report [95.2]. (1995).

[31] Becker RA Wilks AR. “Maps in S”, AT&T Bell Laboratories Statistics Research Report [93.2]. (1993).

[32] Jiang A Zhang TH. Political trust in east and Southeast Asia: the joint effects of education, corruption perception, and urbanization. Int J Public Opin Res. (2021) 33(4):911–26. 10.1093/ijpor/edab008

[33] Sattar A Hassan A Hussain MN Sakhi U Elahi AR. Impact of foreign direct investment on socio-economic development in belt and road countries. Cogent Econ Financ. (2022) 10(1):2143772. 10.1080/23322039.2022.2143772

[34] Schütte S Acevedo PNM Flahault A. Health systems around the world—a comparison of existing health system rankings. J Glob Health. (2018) 8(1):010407. 10.7189/jogh.08.010407 29564084 PMC5857204

[35] Luhar S Timæus IM Jones R Cunningham S Patel SA Kinra S Forecasting the prevalence of overweight and obesity in India to 2040. PLoS One. (2020) 15(2):e0229438. 10.1371/journal.pone.0229438 32092114 PMC7039458

[36] Cai L Hu X Liu S Wang L Wang X Tu H China is implementing the national nutrition plan of action. Front Nutr. (2022) 9:983484. 10.3389/fnut.2022.983484 36071936 PMC9441738

[37] Chen C-L Wu Y-J Yang S-C Wu F-ZJCD. Therapy: new look at the power of zero coronary artery calcium (CAC) in Asian population: a systemic review and meta-analysis. Cardiovasc Diagn Ther. (2024) 14(3):377–87. 38975010 10.21037/cdt-23-474 PMC11223936

[38] Shen Y-W Wu Y-J Hung Y-C Hsiao C-C Chan S-H Mar G-Y Natural course of coronary artery calcium progression in Asian population with an initial score of zero. BMC Cardiovasc Disord. (2020) 20(1):212. 10.1186/s12872-020-01498-x 32375648 PMC7204036

[39] Adab P Pallan M Whincup PH. Is BMI the best measure of obesity? Br Med J. (2018) 360:k1274. 10.1136/bmj.k1274 29599212

[40] Starke G Gille F Termine A Aquino YSJ Chavarriaga R Ferrario A Finding consensus on trust in AI in health care: recommendations from a panel of international experts. J Med Internet Res. (2025) 27:e56306. 10.2196/56306 39969962 PMC11888049

[41] Jolin JR Kwon M Brock E Chen J Kokan A Murdock R Policy interventions to enhance medical care for people with obesity in the United States—challenges, opportunities, and future directions. Milbank Q. (2024) 102(2):336–50. 10.1111/1468-0009.12693 38332667 PMC11176406

