# Supplementary material for: Global, regional, and national burden of cardiovascular diseases attributable to high body mass index from 1990 to 2021
Source: Front Cardiovasc Med. 2025 Sep 12;12:1641689. doi: 10.3389/fcvm.2025.1641689 (PMC12465627; doi:10.3389/fcvm.2025.1641689)
Supplement: Supplementary file 3 [file Datasheet1.pdf]

Supplementary material

Global, regional, and national burden of cardiovascular diseases attributable to high body mass index from 1990 to 2021

Liangtao Yao[1], Wenying Hou[2], Yan Zheng[1,3]* & Guohai Su[1,3]*

[1]*Research Center of Translational Medicine, Central Hospital Affiliated to Shandong First Medical University, Jinan, Shandong, China*

[2]*School of Mathematics and Statistics, Shandong Normal University, Jinan, Shandong, China*

[3]*Department of Cardiovascular Medicine, Central Hospital Affiliated to Shandong First Medical University, Jinan, Shandong, China*

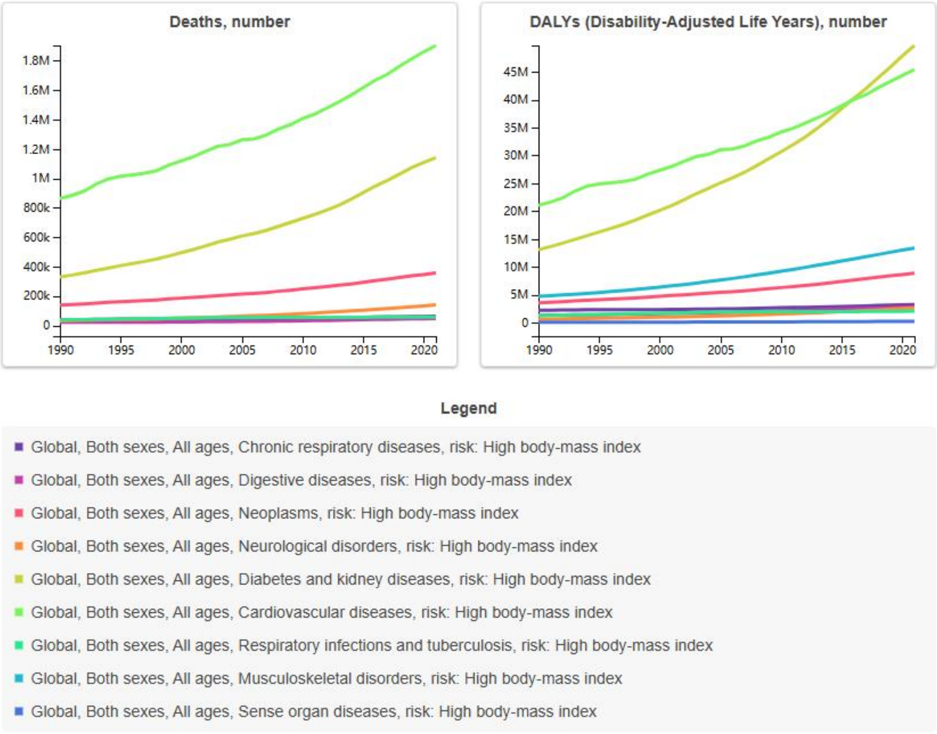

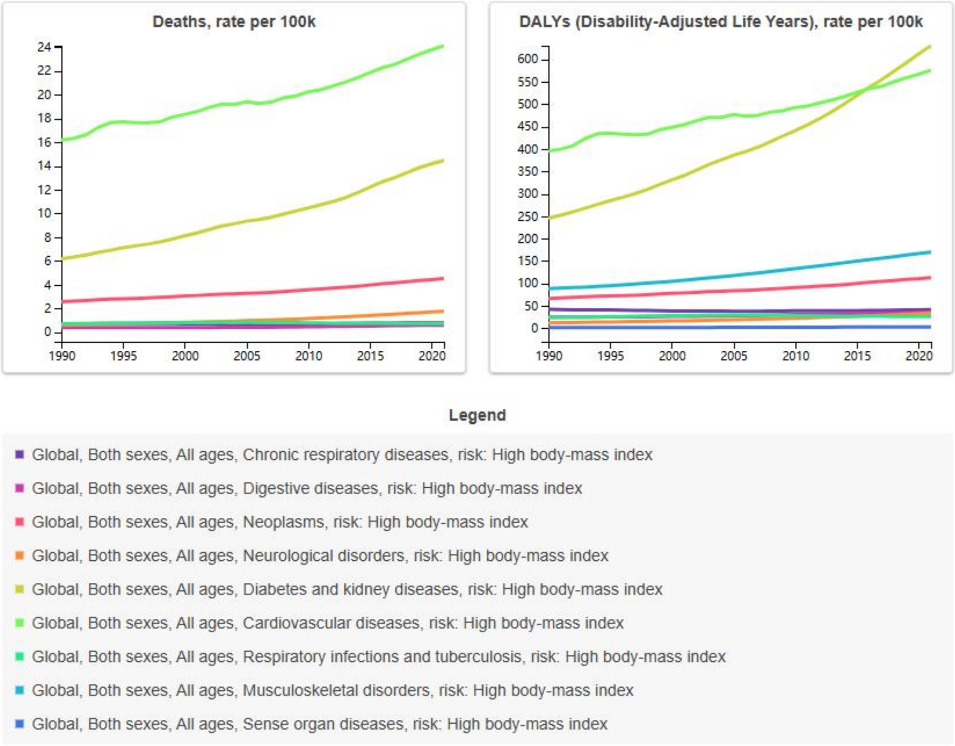

Figure S1. The numbers and rates of deaths and DALYs of top nine diseases attributable to high BMI globally from 1990 to 2021.

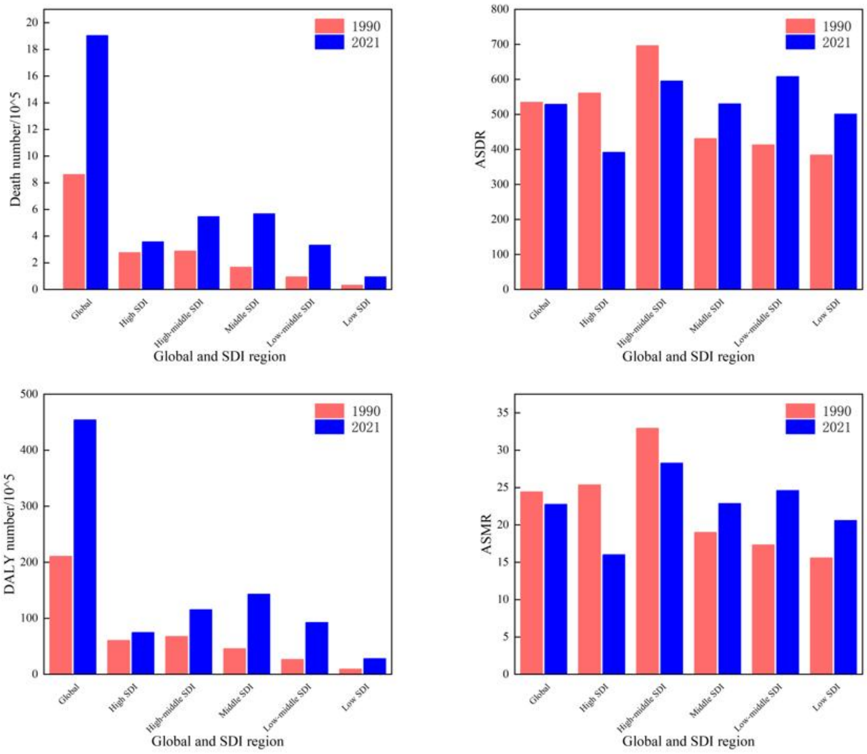

Figure S2. The numbers and rates of deaths and DALYs attributable to high BMI-related CVDs globally and across five SDI regions in 1990 and 2021.

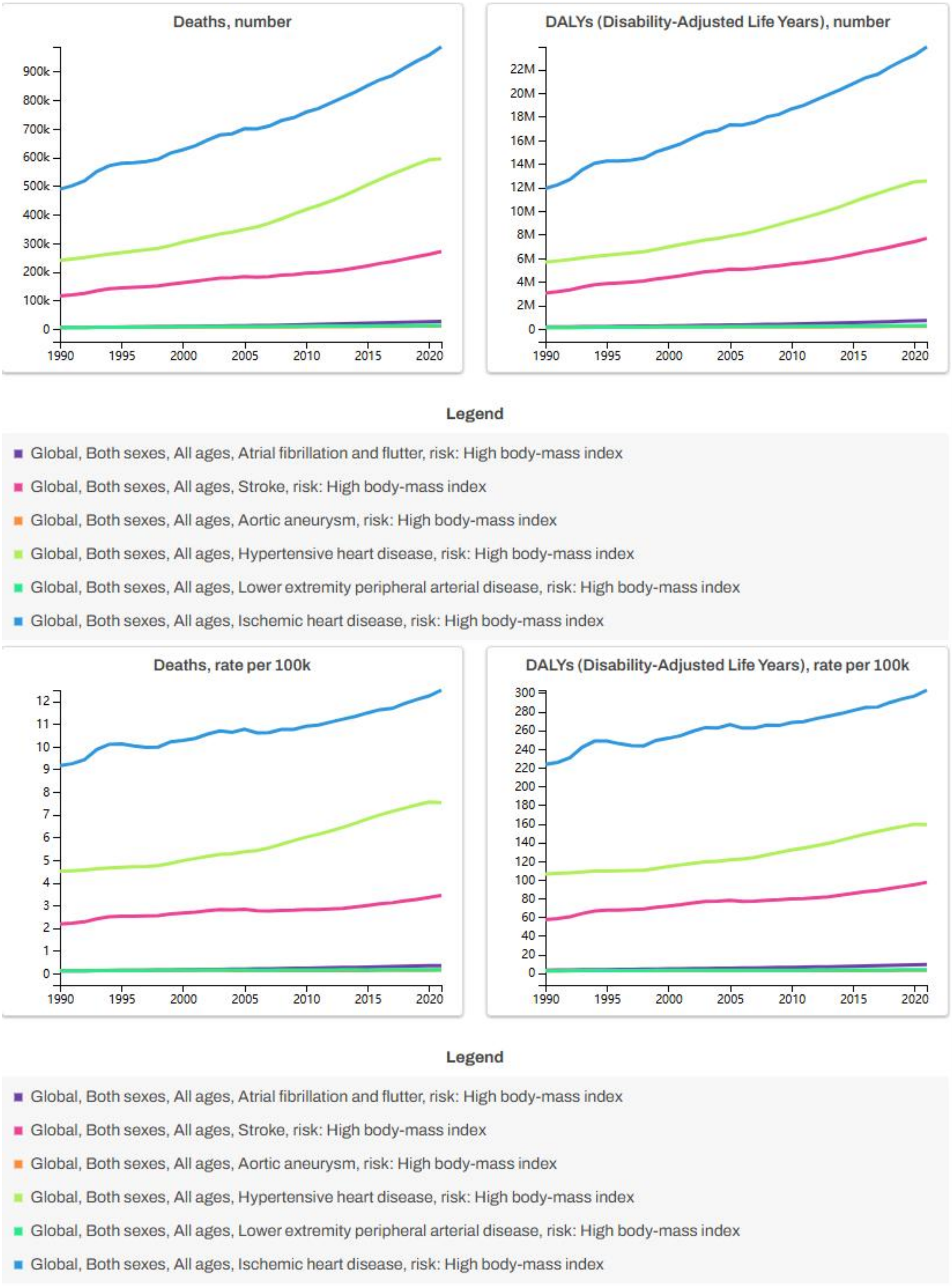

Figure S3. The numbers and rates of deaths and DALYs of six CVD subtypes attributable to high BMI globally from 1990 to 2021.
